# Serum *N*‐glycan profiling as a diagnostic biomarker for the identification and assessment of psoriasis

**DOI:** 10.1002/jcla.23711

**Published:** 2021-01-28

**Authors:** Chengyun Zou, Chenjun Huang, Li Yan, Xin Li, Meng Xing, Bin Li, Chunfang Gao, Haiying Wang

**Affiliations:** ^1^ Shanghai University of Traditional Chinese Medicine Shanghai China; ^2^ Department of Laboratory Medicine The Third Affiliated Hospital of Naval Medical University Shanghai China; ^3^ Department of Clinical Laboratory Yueyang Hospital of Integrated Traditional Chinese and Western Medicine, Shanghai University of Traditional Chinese Medicine Shanghai China; ^4^ Department of Dermatology Yueyang Hospital of Integrated Traditional Chinese and Western Medicine, Shanghai University of Traditional Chinese Medicine Shanghai China

**Keywords:** biomarker, diagnostic model, glycosylation, *N*‐glycan profiling, psoriasis

## Abstract

**Background:**

Glycosylation is an important post‐translational modification of protein. The change in glycosylation is involved in the occurrence and development of various diseases, and this study verified that *N*‐glycan markers might be a diagnostic marker in psoriasis.

**Methods:**

A total of 76 psoriasis patients were recruited. We used Psoriasis Area Severity Index (PASI) scores to evaluate the state of psoriasis, 41 of whom were divided into three subgroups: mild, moderate, and severe. At the same time, 76 healthy subjects were enrolled as a control group. We used DNA sequencer–assisted fluorophore‐assisted carbohydrate electrophoresis (DSA‐FACE) to analyze serum *N*‐glycan profiling.

**Results:**

Compared with the healthy controls, the relative abundance of structures in peaks 5(NA2), 9(NA3Fb), 11(NA4), and 12(NA4Fb) was elevated (*p* < .05), while that in peaks 3(NG1A2F), 4(NG1A2F), 6(NA2F), and 7(NA2FB) was decreased (*p* < .05) in the psoriasis group. The abundance of peak 5 (NA2) increased gradually with the aggravation of disease severity though there was no statistically significant, was probably correlated with the disease severity. The best area under the receiver operating characteristic (ROC) curve (AUC) of the logistic regression model (PglycoA) to diagnose psoriasis was 0.867, with a sensitivity of 72.37%, a specificity of 85.53%, a positive predictive value(PPV) of 83.33%, a negative predictive value(NPV) of 75.58%, and an accuracy of 78.95%.

**Conclusions:**

Our study indicated that the *N*‐glycan–based diagnostic model would be a new, valuable, and noninvasive alternative for diagnosing psoriasis. Furthermore, the characteristic distinctive *N*‐glycan marker might be correlated with the severity gradation of the psoriasis disease.

## Graphical abstract

We used DNA sequencer–assisted fluorophore‐assisted carbohydrate electrophoresis (DSA‐FACE) to analyzed serum N‐glycan profiling. Characteristic changes were revealed in the serum N‐glycome of psoriasis, and we established a diagnostic model based on *N*‐glycan profiling. We also found that the relative abundance of structure in peak 5 (NA2) increased gradually with the increase in disease severity. Our study indicated that the *N*‐glycan–based diagnostic models would be a new, valuable, and noninvasive alternative for diagnosing psoriasis. Furthermore, the characteristic distinctive *N*‐glycan marker might be correlated with the severity gradation of the psoriasis disease.

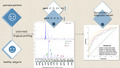

## 1 INTRODUCTION

Psoriasis is a chronic immune‐mediated skin disease with pruritus or painful lesions, and it seriously affects the quality of life,^1^ and the reported prevalence of psoriasis varied from 0.09% in Tanzania to 11.4% in Norway, making psoriasis a serious global problem with at least 100 million people affected worldwide.^2^ In China, a nationwide survey showed that according to Psoriasis Area Severity Index (PASI) scores, 71.1% patients attending hospitals were at moderate and severe state.^3^ The diagnosis of psoriasis was mainly based on the clinical morphological evaluation of the lesion or medical history inquiry. Over the years, many studies focused on finding biomarkers for psoriasis^4^, ^5^, ^6^ and could not make major breakthroughs. A sensitivity and specificity biomarker is urgently needed for early diagnosis of psoriasis.^7^

Recently, increasing evidences have confirmed the N‐linked glycan could be considered as a reliable biomarker for the diseases diagnosis and prognosis.^8^, ^9^ In our previous studies, we identified some *N*‐glycan markers using a capillary‐based electrophoresis called DNA sequencer–assisted fluorophore‐assisted capillary electrophoresis (DSA‐FACE),^10^, ^11^, ^12^ but those researches were mainly focused on cancers. In this study, we firstly analyzed the characteristic changes in serum protein *N*‐glycan in patients with psoriasis, using the modified DSA‐FACE. Compared with the healthy controls, we constructed a diagnostic model PglycoA to value the role of *N*‐glycan markers in psoriasis diagnosis.

## 2 MATERIALS AND METHODS

### 2.1 Patient selection

In total, 76 patients (mean age (SD), 47.82 (16.06) years) with psoriasis were recruited between May 1 and November 30, 2019, at Yueyang Hospital of Integrated Traditional Chinese and Western Medicine, Shanghai University of Traditional Chinese Medicine (hereinafter referred to as Yueyang Hospital) after an informed consent was obtained from each subject included in the study. All the enrolled cases were confirmed by 2 dermatologists. According to PASI score, the disease severity of 41 patients was classified into three subgroups: mild (39.02%, 16/41), moderate (39.02%, 16/41), and severe (14.47%,9/41). Patients with other active skin diseases, systematic treatment with research drugs, biological agents and immunosuppressive agents within 2 months, topical corticosteroids, phototherapy and other treatments within 2 weeks, acute or chronic infection, seriously systemic diseases, history of malignancy, immunodeficiency, or hypersensitivity were excluded from the patient cohort. Seventy‐six age‐ and sex‐matched healthy subjects (mean age (SD), 48.30(14.13) years) were randomly selected from the database established by the third affiliated Hospital of Naval Medical University as a control group (http://206.189.76.64/NGFP/login.php). The collected blood samples were centrifuged at 3000 rpm for 10 minutes and then stored at −80℃ until analysis.

The study protocol was approved by the Research Ethics Committee of Yueyang Hospital.

### 2.2 Laboratory tests

The hematological index, neutrophil count (NEUT#), was determined by automatic cell counter with matching reagents (Sysmex XE‐2100 Cell Counter, Sysmex, Kobe, Japan; Sysmex diagnostic reagents). The biochemical indexes, including total protein (TP), albumin (ALB), total bilirubin (TBIL), alanine aminotransferase (ALT), urea (BUN), creatine (CREA), and uric acid (UA), were tested by automatic biochemical analyzer with matching reagents (Beckman Coulter AU5800 automatic biochemical analyzer, Beckman Coulter, California, American, Beckman Coulter AU diagnostic reagents).

All laboratory tests mentioned above were finished by the Department of Clinical Laboratory, Yueyang Hospital.

### 2.3 Serum protein *N*‐glycan profiling

As described previously,^13^ serum protein *N*‐glycan analyses were performed using DSA‐FACE. In brief, we used peptide‐N‐glycosidase‐F(PNGase F) (New England Biolabs, Boston, Mass) to release the *N*‐glycans presented on the proteins in 2μL of serum, then labeled the *N*‐glycans with fluorescein APTS (8‐amino‐naphthalene‐1,3,6‐trisulfonic acid) (Invitrogen, Carlsbad, Calif.). Sialic acid at the end of the oligosaccharide was removed with arthrobacter ureafaciens sialidase (Roche Bioscience, Palo Alto, Calif.). The processed samples were performed by using capillary electrophoresis–based ABI 3500 Genetic Analyzer (Applied Biosystems, Foster city, Calif.). We use the GeneMapper software (Applied Biosystems) to analyze the 13 obvious *N*‐glycan peaks detected in all serum samples. Each *N*‐glycan structure was described numerically by normalizing its height to the sum of the heights of all peaks.

### 2.4 Statistical analysis

Unless otherwise indicated, all quantitative variables were presented as mean±standard deviation (SD). We used *t* tests, one‐way ANOVA, or nonparametric tests to compare quantitative variables. Pearson's correlation coefficients (Spearman's coefficients of correlation were calculated for ordinal categorical variables) and the associated probabilities (*p*) were used to evaluate the relationship between the parameters. Peaks which the level revealed statistically significant in one‐way ANOVA were selected into a forward stepwise logistic regression analysis to establish the diagnostic models. The individual biomarkers, diagnostic models, and diagnostic performance were evaluated using ROC curve analysis. The sensitivity, specificity, positive predictive value (PPV), negative predictive value (NPV), and accuracy were calculated using optimal cutoff values selected based on the ROC curves. All reported *p* values were 2‐tailed, and *p* values <.05 were evaluated as statistically significant. Statistical analyses were performed using SPSS 26.0 for Windows software (SPSS Inc.).

## 3 RESULTS

### 3.1 Baseline characteristics

In the present study, the main demographic features and laboratory indexes of the patients and healthy subjects are summarized in Table 1. For age and sex, there were no statistical significant differences between both groups. As regards laboratory indexes, except BUN and UA, the values of NEUT#, TP, ALB, TBIL, ALT, and CREA of patient group were significantly different from those of the control group (*p* < .05).

**TABLE 1:** Demographic and clinical characteristics of psoriasis patients and healthy subjects

Characteristics	Control group (n = 76)	Psoriasis group (n = 76)	*P* value
Age (years)	48.30 ± 14.13	47.82 ± 16.06	.843
Gender(M/F)	53/23	54/22	.859
NEUT#(×10^9^/L)	3.56 ± 1.07	4.53 ± 1.55	<.001
TP(g/L)	76.45 ± 3.43	69.36 ± 5.36	<.001
ALB(g/L)	49.11 ± 2.85	42.82 ± 4.75	<.001
TBIL(μmol/L)	15.50 ± 5.82	12.01 ± 4.06	<.001
ALT(U/L)	20.13 ± 12.60	29.90 ± 34.42	.031
BUN (mmol/L)	5.41 ± 1.43	4.94 ± 1.43	.057
CREA(μmol/L)	77.04 ± 13.72	64.82 ± 15.77	<.001
UA(μmol/L)	367.39 ± 78.44	375.59 ± 94.59	.575

Note

Measurement data are expressed as mean ±standard deviation.

Abbreviations: #NEUT, neutrophil count; ALB, albumin; ALT, alanine aminotransferase; BUN, urea; CREA, creatine; TBIL, total bilirubin; TP, total protein; UA, uric acid.

### 3.2 Different profiling patterns of *N*‐glycome in healthy subjects and psoriasis

**FIGURE 1:**
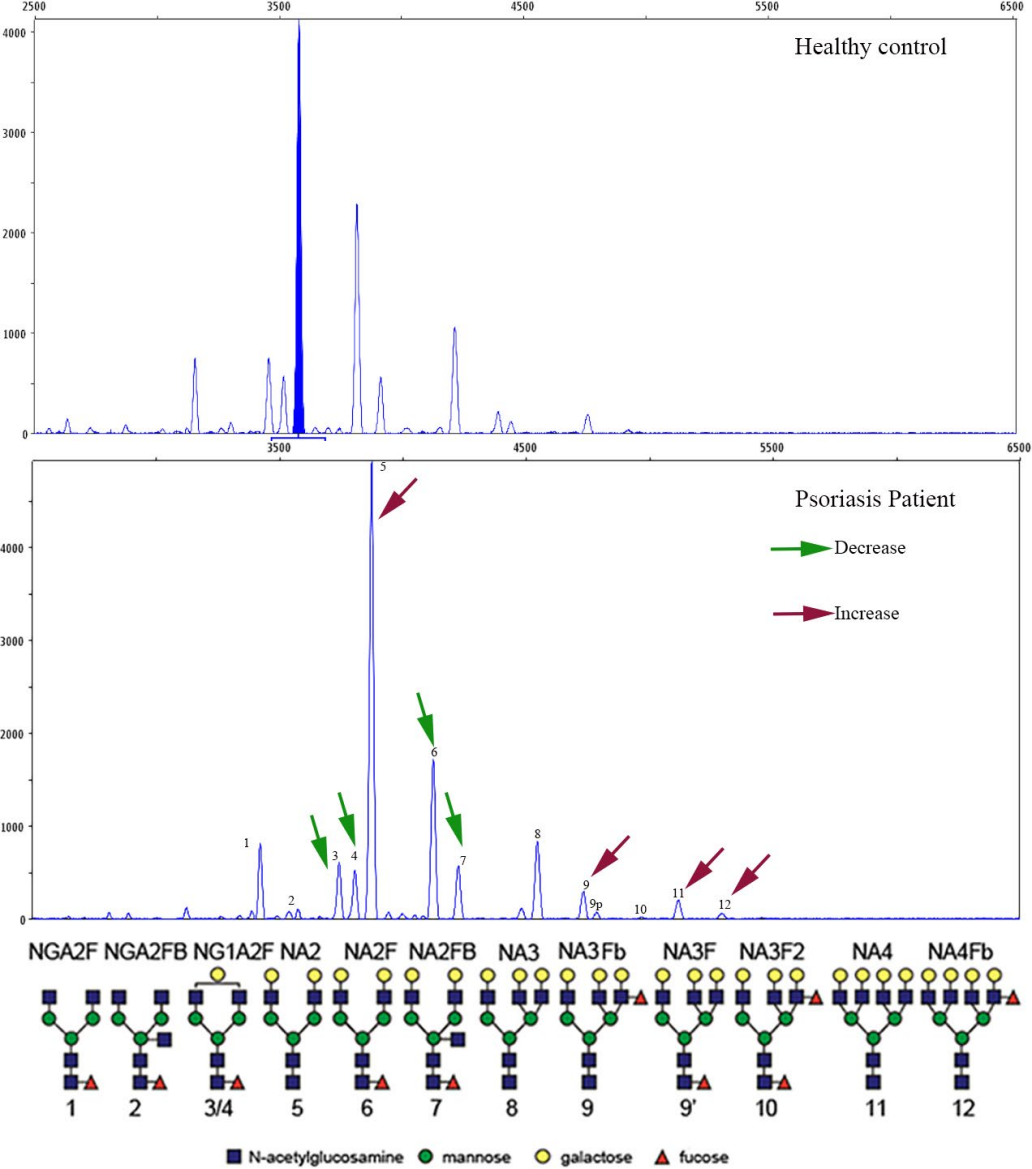
A representative desialylated *N*‐glycan profile extracted from total serum glycoproteins. At least 13 peaks were identified in the psoriasis group and healthy control group. Structures of the *N*‐glycan peaks are shown below the chart and described in Figure 2. The abundance of structures in peaks 5, 9, 11, and 12 was increased, while peaks 3, 4, 6, and 7 were decreased in the patients with psoriasis. Blue square indicates β‐linked N‐acetylglucosamine (GlcNAc); yellow circle indicates β‐linked galactose; red triangle indicates α/β‐1,3/6 linked fucose; green circle indicates α/β‐linked mannose

**FIGURE 2:**
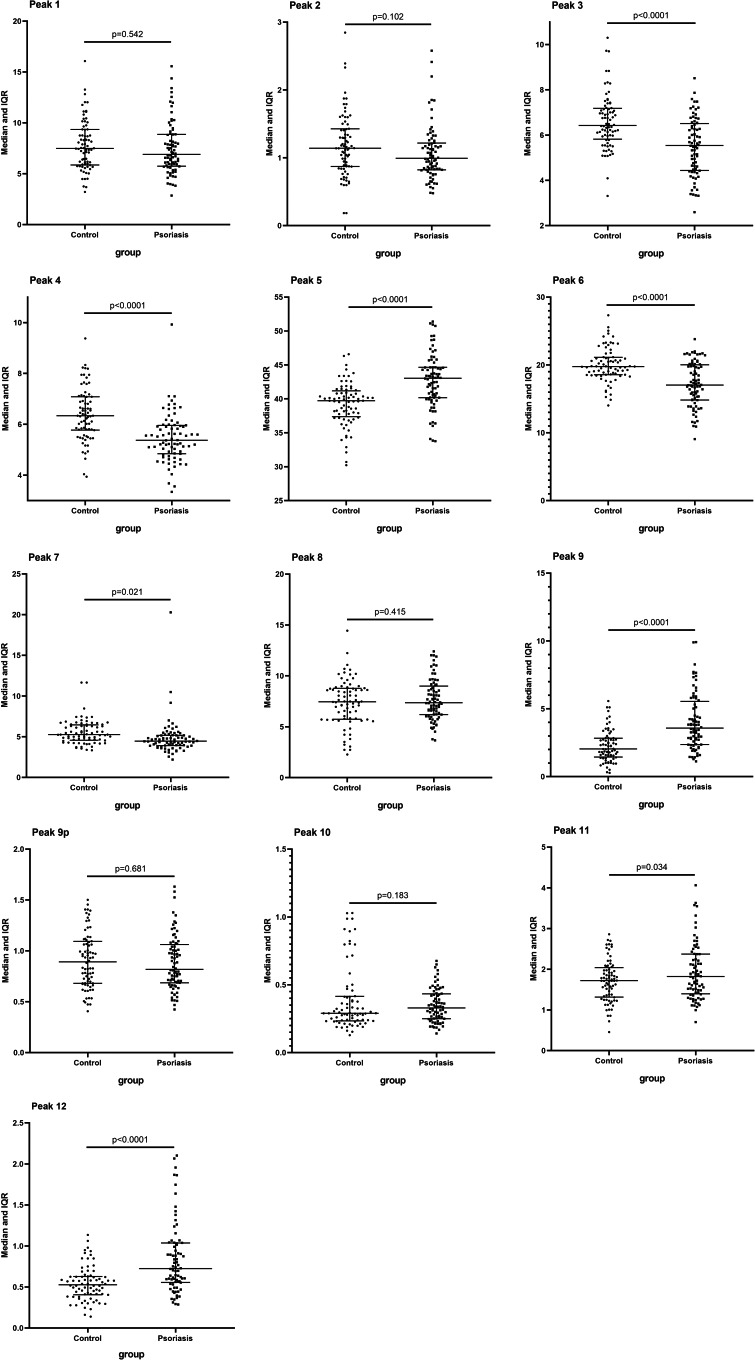
Differences in peaks 3, 4, 5, 6, 7, 9, 11, and 12 between psoriasis group and healthy control group were illustrated in the figure. *p* values <.05 were evaluated as statistically significant

We determined the *N*‐glycan profiling in desialylated sera of patients with psoriasis(n = 76) and healthy subjects(n = 76) using DSA‐FACE. We quantified and statistically compared each peak between 2 groups. Thirteen *N*‐glycan structures in serum were detected in all samples, representative profiling patterns of control and psoriasis patient are shown in Figure 1. The corresponding structure analysis of each *N*‐glycan peak was described previously by Liu et al.^14^ We expressed the average relative abundance of serum *N*‐glycan peaks by using the relative percentage of height to the sum of the heights of all peaks in the profiling. Compared with healthy subjects, the abundance of structures in peaks 3, 4, 5, 6, 7, 9, 11, and 12 revealed a statistically significant difference in patients with psoriasis. Peaks 5(NA2), 9(NA3Fb), 11(NA4), and 12(NA4Fb) were elevated (*p* < .05), while peaks 3(NG1A2F), 4(NG1A2F), 6(NA2F), and 7(NA2FB) were decreased (*p* < .05) in the psoriasis group (Figure 2).

### 3.3 Correlation between *N*‐glycan markers and laboratory indexes

Common laboratory indexes including NEUT#, TP, ALB, TBIL, ALT, BUN, CREA, and UA were chosen. The correlations between 8 *N*‐glycan markers that revealed statistically significant differences in one‐way ANOVA (peaks 3, 4, 5, 6, 7, 9, 11, and 12) and laboratory indexes are listed in Table 2. The decreased *N*‐glycan peaks including peaks 3, 4, and 6 had positive correlation with TP and ALB and negatively correlated with NEUT#. The increased peaks including peaks 5, 9, and 11 had positive correlation with NEUT#, and peaks 5, 9, 11, and 12 negatively correlated with TP and ALB.

**TABLE 2:** Correlations between laboratory indexes and *N*‐glycan markers in psoriasis patients and healthy subjects

Correlations	Peak 3	Peak 4	Peak 5	Peak 6	Peak 7	Peak 9	Peak 11	Peak 12
TP								
*R*	.410	.364	−.483	.408	.167	−.379	−.235	−.389
*P*	<.001*	<.001*	<.001*	<.001*	.058	<.001*	.007*	<.001*
ALB								
*R*	.335	.379	−.340	.341	.060	−.344	−.199	−.359
*P*	<.001*	<.001*	<.001*	<.001*	.501	<.001*	.024*	<.001*
TBIL								
*R*	.197	.165	−.107	.157	−.049	−.100	−.202	−.131
*P*	.025*	.061	.230	.075	.579	.259	.022*	.139
ALT								
*R*	.114	−.069	−.063	.017	−.062	.006	.047	.030
*P*	.197	.437	.481	.845	.486	.947	.598	.739
BUN								
*r*	−.036	.032	.036	.003	.112	−.141	.005	−.181
*P*	.688	.720	.686	.977	.207	.111	.954	.041*
CRE								
*R*	.215	.102	−.068	−.019	−.095	−.103	−.079	−.095
*P*	.014*	.250	.445	.834	.285	.246	.373	.284
UA								
*R*	.045	−.102	.074	−.055	−.085	.148	−.091	.142
*P*	.616	.252	.404	.536	.337	.094	.303	.110
NEUT#								
*R*	−.295	−.186	.295	−.177	−.125	.180	.181	.167
*P*	<.001*	.034*	<.001*	.045*	.157	.042*	.040*	.059

Abbreviations: ALB, albumin; ALT, alanine aminotransferase; BUN, urea; CREA, creatine; NEUT #, neutrophil count; TBIL, total bilirubin; TP, total protein; UA, uric acid.

* Represents significant correlation between laboratory indexes and corresponding N‐glycan biomarkers.

### 3.4 *N*‐glycan biomarkers in different stages of psoriasis

**FIGURE 3:**
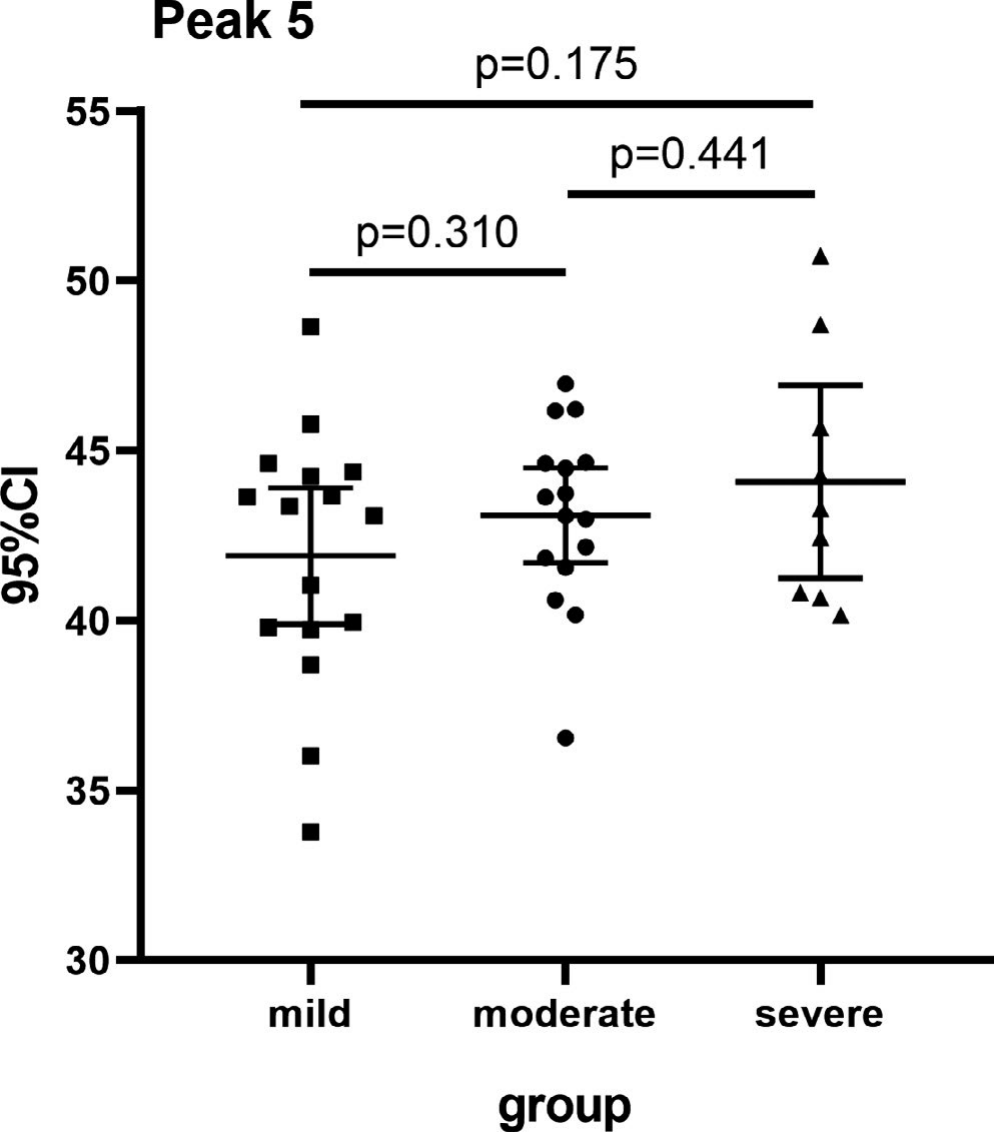
The relative abundance of the structure in peak 5 presented an increasing trend with the progression of disease severity, but there was no significant difference among the three groups. Mild vs moderate (*p* = .310), moderate vs severe (*p* = .441), mild vs severe (*p* = .175)

According to the PASI scores of disease severity index, 41 patients with psoriasis were divided into three subgroups: 16 patients with mild psoriasis (PASI <10), 16 moderate patients (PASI 10 to 20), and 9 severe patients (PASI >20).^15^ The comparative analyses of *N*‐glycan abundance of 13 peaks among the three subgroups are shown in Table 3. Peak 5 (NA2) increased gradually with the aggravation of disease severity, but there was no significant difference (Figure 3) (*p* > .05).

**TABLE 3:** *N*‐glycan biomarkers in three subgroups of psoriasis

Peaks	Mild disease, n = 16		Moderate disease, n = 16		Severe disease, n = 9		*P*	*P*1	*P*2
	Height (Mean±SD)	95% confidence internal	Height (Mean±SD)	95% confidence internal	Height (Mean±SD)	95% confidence internal			
Peak 1	7.26 ± 2.15	6.12–8.40	7.63 ± 2.39	6.35–8.90	6.62 ± 1.70	5.31–7.93	.541	.651	.278
Peak 2	1.10 ± 0.32	0.94–1.27	0.98 ± 0.31	0.82–1.15	0.88 ± 0.21	0.72–1.04	.192	.287	.392
Peak 3	5.03 ± 1.24	4.37–5.69	5.49 ± 1.23	4.83–6.14	5.54 ± 1.22	4.60–6.47	.483	.301	.919
Peak 4	5.76 ± 1.48	4.97–6.55	5.64 ± 0.80	5.21–6.06	5.70 ± 0.69	5.17–6.23	.955	.781	.855
Peak 5	41.91 ± 3.78	39.90–43.93	43.10 ± 2.63	41.70–44.50	44.10 ± 3.70	41.26–46.94	.286	.310	.441
Peak 6	17.20 ± 3.07	15.56–18.83	17.29 ± 3.63	15.35–19.22	17.97 ± 2.74	15.86–20.08	.836	.941	.629
Peak 7	5.72 ± 3.95	3.62–7.83	4.59 ± 1.78	3.64–5.54	4.66 ± 0.92	3.95–5.37	.462	.304	.912
Peak 8	8.21 ± 2.34	6.96–9.46	7.04 ± 1.59	6.19–7.88	6.99 ± 1.20	6.08–7.91	.153	.109	.946
Peak 9	3.83 ± 1.77	2.88–4.77	4.31 ± 2.71	2.87–5.76	3.95 ± 1.97	2.43–5.46	.817	.551	.725
Peak 9p	0.90 ± 0.34	0.72–1.08	0.81 ± 0.23	0.68–0.93	0.86 ± 0.27	0.65–1.07	.659	.374	.599
Peak 10	0.36 ± 0.14	0.28–0.43	0.33 ± 0.08	0.29–0.38	0.36 ± 0.15	0.24–0.47	.863	.602	.643
Peak 11	1.95 ± 0.68	1.59–2.31	1.86 ± 0.70	1.49–2.24	1.61 ± 0.34	1.35–1.87	.429	.719	.321
Peak 12	0.78 ± 0.41	0.56–1.00	0.93 ± 0.56	0.64–1.23	0.77 ± 0.36	0.49–1.05	.583	.391	.435

Note

Measurement data are expressed as means ±standard deviations; *P*1: comparison between mild and moderate psoriasis; *P*2: comparison between moderate and severe psoriasis.

### 3.5 Construction and assessment of diagnostic model based on *N*‐glycan markers for psoriasis from healthy subjects

**FIGURE 4:**
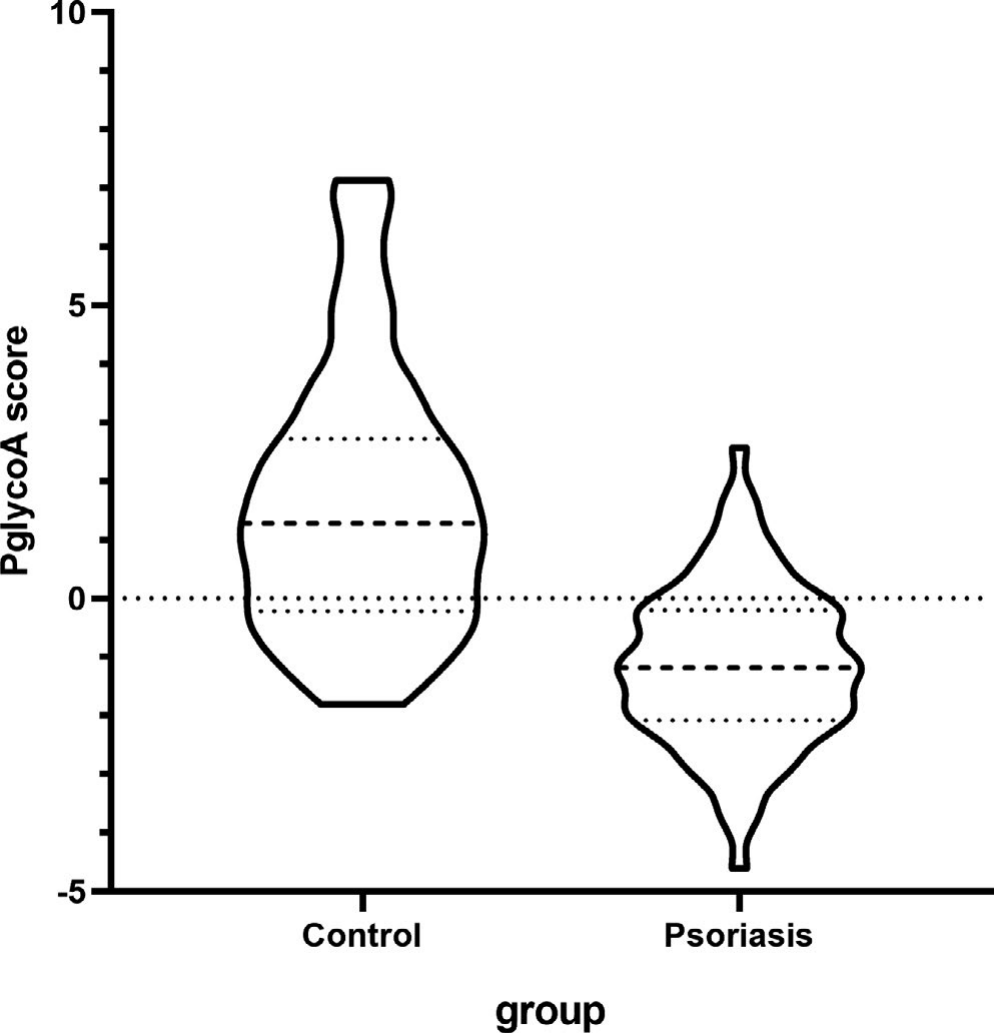
Violin plot of PglycoA score calculated from result of logistic regression model (PglycoA score =−3.047–0.486*Peak 4 + 1.834*Peak 9 + 1.897*Peak 11–4.433*Peak 12)

**FIGURE 5:**
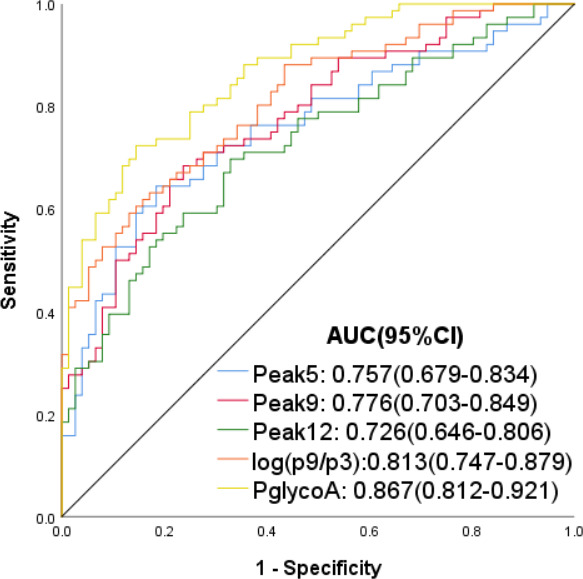
ROC curve for the diagnosis of psoriasis, including peak 5 (0.757, 95%CI: 0.679–0.834), peak 9(0.776, 95%CI: 0.703–0.849), peak 12(0.726, 95%CI: 0.646–0.806), log(p9/p3)(0.813, 95%CI: 0.747–0.879), and diagnostic model PglycoA(0.867, 95%CI: 0.812–0.921)

We found a single agalacto core,1–6,fucosylated biantennary glycan (NG1A2F, peak 3) was decreased in psoriasis, while a branching a‐1,3‐fucosylated triantennary glycan (NA3Fb, peak 9) was increased in psoriasis. To take advantage of the differences, we used log ratio of these 2 structure abundances log(p9/p3) to specify psoriasis and healthy subjects. In addition, logistic regression analysis was used to estimate psoriasis *N*‐glycan alterations, based on logistic regression coefficients, we evaluated odds ratios for each of the independent peaks, which revealed statistically significant in one‐way ANOVA. The mathematic formula PglycoA was constructed to differentiate psoriasis from healthy subjects, PglycoA=−3.047–0.486*Peak 4 + 1.834*Peak 9 + 1.897*Peak 11–4.433*Peak 12(Table 4). The PglycoA score calculated from result of logistic regression model was indicated in Figure 4. The diagnostic efficacies of peak 5(NA2), peak 9(NA3Fb), peak 12(NA4Fb), log(P9/P3), and PglycoA in the diagnosis of psoriasis were evaluated by characterizing the AUC, respectively (Figure 5). Compared with peak 5(AUC=0.757), peak 9(AUC=0.776), peak 12(AUC=0.726), and log(p9/p3)(AUC=0.813), PglycoA(AUC=0.867) was more effective to distinguish psoriasis patients from normal controls. The sensitivity, specificity, PPV, NPV, and accuracy for the prediction of psoriasis by peak 5, peak 9, peak 12, log (P9/P3), and PglycoA are listed in Table 5. ROC curve analysis selected the optimal cutoff values for PglycoA (0.079) with a sensitivity of 72.37%, a specificity of 85.53%, a PPV of 83.33%, a NPV of 75.58%, and an accuracy of 78.95%.

**TABLE 4:** Parameter estimates for the logistic model for psoriasis

Variable	Coefficient, 95% CI	SE	χ2	*P*	OR
Peak 4	−0.486(−1.142 to −0.169)	0.222	4.788	.029	0.615
Peak 9	1.834(1.065 to 3.155)	0.477	14.769	<.001	6.26
Peak 11	1.897(1.045 to 3.127)	0.509	13.906	<.001	6.663
Peak 12	−4.433(−9.681 to −0.392)	2.224	3.972	.046	0.012
Constant	−3.047(−6.956 to 1.756)	2.004	2.311	.128	0.048

Abbreviations: OR, odds ratio; SE, standard error.

**TABLE 5:** Cutoff value, sensitivity, specificity, PPV, NPV, and AUC of *N*‐glycan biomarkers in the diagnosis of psoriasis

**Peaks**	**Cutoff Value**	**Actual Status**			**Sensitivity %, 95% CI**	**Specificity %, 95% CI**	**PPV %, 95% CI**	**NPV %, 95% CI**	**Accuracy %, 95% CI**	**AUC 95% CI**
		**Test**	**P+**	**P‐**						
Peak 5	41.550	P+	49	14	64.47	81.58	77.78	69.66	73.03	0.757
		P‐	27	62	(53.46–75.48)	(72.66–90.50)	(67.22–88.33)	(59.92–79.40)	(65.89–80.16)	(0.679–0.834)
Peak 9	2.843	P+	52	18	68.42	76.32	74.29	70.73	72.37	0.776
		P‐	24	58	(55.13–76.51)	(66.54–86.1)	(63.79–84.78)	(58.13–78.34)	(65.18–79.56)	(0.703–0.849)
Peak 12	0.590	P+	53	26	69.74	67.11	67.95	68.92	68.42	0.726
		P‐	23	51	(59.17–80.30)	(55.43–77.04)	(56.50–77.68)	(58.12–79.71)	(60.50–75.45)	(0.646–0.806)
Log(P9/P3)	−0.264	P+	48	13	63.16	82.89	78.69	69.23	73.03	0.813
		P‐	28	63	(52.06–74.25)	(74.23–91.56)	(68.11–89.26)	(59.57–78.90)	(65.89–80.16)	(0.747–0.879)
PglycoA	0.079	P+	55	11	72.37	85.53	83.33	75.58	78.95	0.867
		P‐	21	65	(62.08–82.65)	(77.43–93.62)	(74.10–92.57)	(66.32–84.85)	(72.39–85.50)	(0.812–0.921)

Abbreviations: CI, confidence interval; NPV, negative predictive value; P, psoriasis; PPV, positive predictive value.

## 4 DISCUSSIONS

Psoriasis is a chronic, recurrent, inflammatory skin disease. It is characterized by keratinocytes proliferate excessively, inflammatory cell infiltration and neovascularization, with a strong genetic predisposition and immune‐mediated pathogenic trait.^16^, ^17^ In China, a nationwide survey in 1984 revealed that the prevalence of psoriasis was 0.12%,^18^ it means that psoriasis patients in China are estimated to be 1.6 million(1.33 billion in 2010 census), but the most recent study conducted in purposively selected six cites in 2012 revealed that it was 0.47%,^19^ indicating a slight increase compared with the prevalence in 1984. Patients with psoriasis suffer from both psychological and physical pain, due to the wrong and delated diagnosis, inappropriate treatment, and social prejudice, which can be avoided through early intervention.^2^, ^20^

Nowadays, the diagnosis of psoriasis depends on the morphology, boundary, and distribution of skin lesion. Although histopathological analysis of skin biopsy contributes efficient clinical identification, it is difficult to identify psoriasis resulting from unconspicuous pathologic changes.

In the early years, studies showed that pro‐inflammatory cytokines, including tumor necrosis factor, interferon‐γ, interleukin‐6, interleukin‐8, interleukin‐12, interleukin‐18,^21^ and unspecific inflammation markers, for example, C‐reactive protein and haptoglobin,^22^ increased in serum of psoriasis patients. However, most of them cannot provide sufficient sensitivity or specificity. Although more and more studies have shown that some hormones, proteins, and lipids present differences in psoriasis patients and healthy subjects, it is difficult to differentially diagnose psoriasis from other diseases, and it is also not clear whether quantitative numerical values could be used to explain the correlations with disease progression.^23^, ^24^, ^25^ So they are still unsuitable for objective assessment of disease status.

Glycosylation is an important post‐translational modification of proteins, which plays a significant role in cell adhesion, molecular transport and clearance, receptor activation, signal transduction, and immune cell trafficking.^26^, ^27^, ^28^, ^29^ Glycomics is widely used in the study of genetic background, pathogenesis, and diagnostic markers of diseases.^30^ In the 1980 s, the researchers found that the abnormal growth of psoriatic epidermis might be related to the fucose‐labeled glycoproteins from keratinocytes in psoriasis lesions.^31^ In terms of psoriasis, in the 1990 s, it was confirmed that the carbohydrates (types 2 and 3 chain H and T) were expressed only at the early stage of cell maturation, and T‐structure biosynthetic precursors (Tn and sialyl‐Tn), which appeared in psoriatic skin, are not expressed in normal skin.^32^

As an *N*‐glycan profiling detection technique, DSA‐FACE is simple and efficient due to its high sensitivity, throughput, and speed, and it has been widely applied to detect *N*‐glycan in cancers and other diseases.^33^, ^34^, ^35^ Previously, we used this technology and successfully established disease diagnostic models; here, we used DSA‐FACE to analyze N‐linked glycan and to seek the specific *N*‐glycan profiling pattern occurring in psoriasis. The results indicated that there were significant differences in *N*‐glycan profiling patterns between psoriasis group and healthy group. The data of the laboratory indexes suggested the psoriasis patients might be in a state of protein nonhomeostasis and inflammation.^36^ Our study also demonstrated that *N*‐glycan markers were correlated well with clinical indexes, which showed that the decreased *N*‐glycan peaks had positive correlation with TP and ALB and negatively correlated with NEUT#, and the increased peaks had positive correlation with NEUT#, and negatively correlated with TP and ALB. Previous studies had showed that there were different proteins in serum between patients with psoriasis and healthy subjects.^37^, ^38^ The disorder of cell metabolism in patients with psoriasis may affect the proteomic profile.^39^ As we know that the serum *N*‐glycan profiling comes from serum N‐glycoproteins. In recent years, people had successfully found a series of new potential glycobiology tumor markers, by using lectin‐related research technology, such as alpha‐fetoprotein‐L3,^40^ Golgi Protein 73,^41^ haptoglobin,^42^ and alpha‐acid glycoprotein.^43^ Recently, It was reported that increased glycan branches and fucose content of haptoglobin (Hp) were associated with psoriasis.^44^, ^45^ The ability of keratinocytes in psoriasis skin lesions to synthesize Hp is significantly enhanced,^45^ may be secreted into the skin,^46^ which can inhibit the ability to activate antigen‐presenting cells’ functional differentiation of Langerhans cells (LCs).^47^ Haptoglobin‐synthesizing keratinocytes might play a role of negative feedback regulation in the pathogenesis of psoriasis. The content of core‐fucosylated *N*‐glycan enhances significantly in the process of tissue malignant evolution, considerably higher than in normal tissues.^48^ Alpha‐(1,6)‐fucosyltransferase 8(FUT8) is implicated in the pathogenesis of several malignancies, Kelel M et al found the elevated FUT8 expression in the lesional epidermis is implicated in the development of psoriasis phenotypes, and FUT‐8 promotes the proliferation of epidermal keratinocytes through the EGFR/Akt signaling pathway by controlling the expression of cyclin, which highly expressed in the epidermal cells of patients with psoriasis and is related to the severity of the disease.^49^ In the future, we will enrich the core fucoglycosylated serum total protein in healthy subjects and psoriasis patients with lens culinaris agglutinin (LCA) specifically binding core fucoglycosylated glycans. Isobaric Tag for relative and absolute quantitation‐time of flight(iTRAQ‐TOF) will be used to further analyze the protein differences between the samples and to explore the specific glycoprotein markers that could contribute diagnosis of psoriasis, and fucosylated haptoglobin will be focused as one of the specific target glycoproteins. Meanwhile, we are also going to investigate the relevant regulatory molecules concerning the *N*‐glycan markers in the further study.

According to the peaks with statistical differences between the psoriasis patients and the healthy control, we constructed 2 diagnostic models: log(p9/p3) and PglycoA. Both models demonstrated satisfactory diagnostic performances, and PglycoA (AUC=0.867) was more effective than log(p9/p3) (AUC=0.813). We also found that peak 5 increased gradually with the aggravation of disease severity and might be associated with the development of psoriasis, but there was no significant difference among psoriasis groups.

There existed a limitation of this study, which is that not all the enrolled psoriasis subjects evaluated the state of psoriasis, but it does not affect the conclusions; in the future, we would expand the size of patients to further validate whether our diagnosis model could actually effectively be used in clinical diagnosis of psoriasis.

In summary, we analyzed the specific *N*‐glycans in the serum of psoriasis patients for the first time using modified DSA‐FACE. Our study indicated that the structure abundances of 4 *N*‐glycan in serum were significantly increased in psoriasis patients compared with 76 corresponding healthy subjects, while 4 *N*‐glycan structure abundances were obviously decreased. Moreover, peak 5 was increased gradually with the aggravation of disease severity. The logistic regression model PglycoA might be a promising biomarker in early diagnosis of psoriasis, and peak 5 might be exerted in progression monitoring. The *N*‐glycan profiling in serum might contribute diagnosis of psoriasis timely in the early stages.

## CONFLICT OF INTEREST

The authors declare that there are no conflicts of interest,

## AUTHOR'S CONTRIBUTIONS

Chengyun Zou and Chenjun Huang make the same contribution to this article.

## Data Availability

All data included in this study are availability upon request by contact with the corresponding author.
